# Role of dexamethasone in the para-vertebral block for pediatric patients undergoing aortic coarctation repair. randomized, double-blinded controlled study

**DOI:** 10.1186/s12871-018-0637-y

**Published:** 2018-11-30

**Authors:** Amany H. Saleh, Passaint F. Hassan, Mohamed Elayashy, Hamza M. Hamza, Mona H. Abdelhamid, Mai A. Madkour, Pierre Z. Tawadros, Heba Omar, Mohamed M. Kamel, Marwa Zayed, Mohamed Helmy

**Affiliations:** 1 Department of Anesthesia , Surgical Intensive Care and Pain Management, Faculty of Medicine, Cairo University, Cairo, Egypt; 2 el Haram, Giza, Egypt

**Keywords:** Aortic coarctation, Ultrasound guidance, Paravertebral block, OPS

## Abstract

**Background:**

Surgery for aortic coarctation requires special care during anesthesia due to severe pain during the lateral thoracotomy incision, intraoperative hemodynamic instability and the need for large doses of intra- and postoperative analgesics and vasodilators. Additionally, the postoperative care of patients is very important.

**Aims:**

We aimed to compare ultrasound-guided paravertebral block performed using bupivacaine alone and bupivacaine with dexamethasone in terms of the intra- and postoperative analgesic requirements and hemodynamics, postoperative complications and ICU stay.

**Study design:**

This was a prospective, randomized, controlled, double-blinded study.

**Methods:**

Fifty patients aged four to 12 months scheduled for aortic coarctation surgery were randomly divided into two equal groups (*n* = 25). Patients in group D (dexamethasone) received 0.5 mg/kg bupivacaine 0.25% mixed with 0.1 mg/kg dexamethasone diluted with isotonic saline and those in group C (control) received 0.5 mg/kg bupivacaine 0.25% diluted with isotonic saline (total volume 15 ml in each group). Intraoperative fentanyl consumption and hemodynamics (heart rate, arterial blood pressure) at baseline, 1 min after induction, at skin incision, after 30 min, after clamping, after declamping and at the end of the surgery were recorded, along with the objective pain score (OPS) immediately postoperatively and at 4 h, 8 h, 12 h and 24 h postoperatively and the time to the first request for pethidine. The intra- and postoperative vasodilator doses, time to extubation, ICU stay duration and postoperative complications were also recorded.

**Results:**

The postoperative OPS was significantly lower at 12 and 24 h in group D than in group C. The time to the first request for analgesia was significantly longer in group D than in group C (3.9 ± 2.23 vs 8.6 ± 0.69). Additionally, the time to extubation was significantly shorter in group D.

**Conclusion:**

The use of dexamethasone as an adjuvant in ultrasound-guided paravertebral block in paediatric patients undergoing surgery for aortic coarctation increased the duration of postoperative analgesia with a prolonged time to the first request for analgesics It was also associated with a decreased incidence of postoperative complications.

**Trial registration:**

Trial registration number: NCT03074773. (Prospectively registered). The initial registration date was 9/3/2017.

## Background

Thoracotomy pain is severe, especially in paediatric patients. The severity arises from the surgical wound site, damage to the ribs, chest wall separation, disruption of the pleura and intercostal nerves and the site of intercostal tube insertion [1, 2]. Postoperative pain management following thoracotomy represents a major challenge, especially in children; additionally, improper postoperative analgesia can compromise respiratory function, prolong the hospital stay, and increase the cost of treatment [3].

Coarctation of the aorta is one of the most common congenital anomalies, accounting for 5–8% of congenital cardiac anomalies. Most of these patients present with hypertension and variable degrees of heart failure [4]. Aortic coarctation requires intervention by either surgery or balloon angioplasty [5]. Anaesthetic management during surgery for aortic coarctation requires special care due to the intraoperative hemodynamic instability related to clamping and declamping of the aorta. Additionally, postoperative patient care is very important as it concerns controlling residual hypertension and maintaining adequate organ perfusion while avoiding problems related to mechanical ventilation, such as laryngeal oedema, tube kinking or obstruction by mucus plugs and accidental extubation. Additionally, the excessive use of sedatives to facilitate endotracheal tube tolerance may delay postoperative extubation [6].

Paravertebral block is an effective analgesic technique used in various types of surgery and the treatment of trauma and chronic pain. Its analgesic effect results from the penetration of a local anaesthetic into the spinal nerve, including the dorsal ramus, ramus communicans and sympathetic chain, thus eliminating the cortical response to thoracic stimulation. The addition of steroids may exert a membrane-stabilizing effect on C fibres and can produce more analgesia. Additionally, steroids have a direct anti-inflammatory effect, resulting in a prolonged effect when used on conjunction with local anaesthetics. The pharmacodynamics and pharmacokinetics of drugs when administered in a regional nerve block are difficult to explain, which might be due to a combined somatosensory and sympathetic blockade. The closer the nerve damage is to the injected local anaesthetics and steroids, the more beneficial the effect of both the anaesthetics and steroids [7].

Paravertebral block decreases postoperative pain and reduces opioid consumption, in turn improving postoperative pulmonary function and potentially enabling early extubation [8]. Moreover, when used unilaterally, paravertebral block results in less sympathectomy and consequently much fewer hemodynamic side effects than thoracic epidural anaesthesia. Furthermore, there is no motor block [9].

Ultrasound guidance can dramatically increase the success rate of the procedure and minimize the incidence of technical complications.

In this study, we aimed to compare ultrasound-guided paravertebral block using bupivacaine alone and bupivacaine with dexamethasone in terms of the intra- and postoperative analgesic requirements and hemodynamics, postoperative complications and ICU stay duration in children undergoing surgery for aortic coarctation.

## Patients and methods

After approval was granted by our institutional ethical committee and the study was registered as a randomized, double-blinded clinical trial (registry ID: NCT03074773), 50 patients aged four to 12 months were scheduled for aortic coarctation surgery in the paediatric cardiothoracic surgery theatre of Abu el Reesh hospital. Patients with other cardiac anomalies (e.g ventricular septal defect), airway abnormalities, heart failure, endocrine disorders, a history of convulsions, neurological disorders, hepatic, renal or neuromuscular disease, coagulopathy, a history of hyperthermia, infection at the site of the block or a family history of hypersensitivity were excluded from the study. Patients were allocated to the study groups using a computer-generated random list, and the group assignments were sealed in sequentially numbered opaque envelopes that were opened after the induction of anaesthesia.

The anaesthesiologist interviewed the guardians, obtained informed consent, examined the patients, and checked all routine investigations, which included a complete blood count (CBC), coagulation profile, liver function tests, renal function tests, blood grouping, chest X-ray, recent echocardiography and angiography if available.

Children in both groups were premedicated with midazolam 0.3 mg/kg IM and atropine 0.02 mg/kg IM 10 min prior to the induction of anaesthesia. Then, they transferred to the OR where they were placed on a warming mattress; all non-invasive monitors were applied (electrocardiography (ECG), pulse oximetry and non-invasive BP), and the baseline heart rate (HR) and blood pressure (BP) were recorded. In both groups, anaesthesia was induced using 3% sevoflurane, followed by peripheral cannula insertion and fentanyl 3 μg/kg. Atracurium 0.5 mg/kg was administered to facilitate endotracheal intubation and was repeated intraoperatively as required to maintain muscle relaxation. Anaesthesia was maintained using sevoflurane 0.3–1.5% in an oxygen-air mixture (1:1 ratio) and atracurium 0.5 mg/kg/hr. An external jugular venous line was inserted on the nondependent side and an arterial line was inserted on the dependent side. HR and invasive arterial BP (ABP) values were recorded after induction. A nasopharyngeal temperature probe was inserted, as was a urinary Foley catheter to monitor renal perfusion and urine output.

The patients were divided into two groups according to the type of drug injected. Patients in group D(dexamethasone) received 0.5 mg/kg bupivacaine 0.25% mixed with 0.1 mg/kg dexamethasone diluted with isotonic saline (total volume 15 ml), and those in group C (control) received 0.5 mg/kg bupivacaine 0.25% diluted with isotonic saline (total volume 15 ml). Syringes of local anaesthetics were prepared by the nurse, and the investigator was blinded to the type of drug injected. Ultrasound-guided paravertebral block was applied in both groups after the induction of anaesthesia.

All patients were tilted onto their left side (lateral position) while the ultrasound-guided paravertebral block was performed under completely aseptic conditions. The ultrasound probe was covered by a sterile gel film. The anatomy was examined by ultrasonography at 5–6 Hz with a35 mm linear probe and a SonoSite M-turbo system (Fijufilm SonoSite, Inc., USA). The ultrasound transducer was placed in a transverse position, and the probe was moved superiorly and inferiorly to confirm the correct position (T6). The transverse process was visualized medially with the pleura dipping under the inferolateral aspect. The internal intercostal membrane, which was continuous with the superior costotransverse ligament, was generally observed as a thin radio-opaque line extending from the transverse process, creating a wedge-shaped pocket representing the thoracic paravertebral space. A22 g needle was generally more easily visualized with this approach because the angle of reflectance was not as acute as with the longitudinal parasagittal technique. If the needle was difficult to see when clearly in the plane, tissue dissection with small aliquots of normal saline or local anaesthetic was used as previously described to assist in confirming the needle placement.

The needle tip was placed in the hypoechoic triangle space formed by the acoustic shadow underneath the transverse process medially, the antero-lateral pleura and the lower border of the intercostal muscle. Correct positioning of the needle was confirmed by anterior displacement of the pleura upon the injection of the small bolus of saline or local anaesthetic.

Once confirmed, when the needle pierced the internal intercostal membrane, and after careful aspiration to demonstrate the absence of air or blood, the required volume of local anaesthetic was injected [10], and left thoracotomy was performed. Dissection commenced towards the narrowed segment of the aorta. Heparin was administered prior to clamping (100 IU/kg); then, the clamps were applied. Vasodilator therapy was instituted using nitroglycerine if the systolic pressure > 85 mmHg in infants or > 95 mmHg in children. During the operation, if the HR exceeded the baseline by 20%, an additional dose of fentanyl was administered (1–2 μg/kg) as rescue analgesia. The repair was performed using an end-to-end, extended end-to-end or subclavian flap technique.

After completion of the repair, the clamps were removed, protamine sulphate was administered at 1 mg/kg for each 100 IU/kg heparin to reverse the heparin action, and haemostasis was achieved. The closure was performed in layers after expanding the collapsed lung.

After completion of the surgery, inhalational anaesthetics were stopped as were the muscle relaxers. The patient was then transferred to the ICU. The pain intensity was assessed using the objective pain score (OPS) by a person who was blinded to the treatment [11], and pethidine was administered as rescue analgesia to maintain the OPS below five.

Our primary endpoint was the 12-h postoperative OPS, and the secondary endpoints were the intraoperative fentanyl consumption, the hemodynamic parameters (HR, ABP) at baseline, 1 min after induction, at skin incision, after 30 min, after clamping, after declamping and at the end of surgery, the postoperative OPS immediately postoperative and at 4 h, 8 h, 12 h and 24hpostoperatively, and the time to the first request for pethidine. Other recorded measurements included the patient characteristics, intraoperative and postoperative vasodilator doses, time to extubation, ICU stay duration and postoperative complications (nausea, vomiting, respiratory depression, bradycardia, and hypotension).

### Statistical analysis

According to a previous study [12] showing a mean ± SD VAS score of patients who received paravertebral block using bupivacaine and dexamethasone of 2.7 ± 1.93, assuming a 30% difference between the two groups with a power of 80% and an alpha error of 0.05, the required sample size for each group was 20, as determined using G Power software (version3.1.3 Heinrich-Heine-Universität Düsseldorf, Düsseldorf, Germany). This number was increased to 25 per group to account for possible dropouts. The data were summarized and analysed using SPSS software (SPSS Statistics for Windows, Version 17.0., SPSS, Inc., USA). Continuous data are presented as the mean ± SD. Nonparametric data are presented as the median and range. Comparison of the means of the study groups was performed using Student’s t-test and repeated measures ANOVA. Categorical data are presented as frequencies and were compared using the chi-square (× 2) test. In all statistical tests, the level of significance was fixed at 5%. A *p*-value < 0.05 was considered to indicate a significant difference.

## Results

Fifty patients were enrolled in the study and divided evenly into two groups, each with 25 patients. The patient characteristics are presented in Table 1 and showed no significant difference between the groups.

**Table 1:** Patients’ characteristics

	Control (*n* = 25)	Dexamethasone (*n* = 25)	*P* value
Age mean (SD)	8.3(2.16)	8.18(2.27)	.904
Sex (Male)	52%	44%	.835
Weight mean (SD)	8.5(1.84)	8.32(1.86)	.825
Duration of surgerymean (SD)	3.25(0.26	3.22(0.26)	.845

There was a significant difference between the two groups regarding the time to the first request for analgesia and the time to extubation. There was no difference regarding intraoperative fentanyl consumption. The intra- and postoperative vasodilator doses were lower in the dexamethasone group, but there were no significant differences, as presented in Table 2.

**Table 2:** Mean (SD) of block duration, intraoperative fentanyl consumption, time to first request of analgesia, intraoperative and postoperative vasodilator and time to extubation

	Control (*n* = 25)	Dexamethasone (*n* = 25)	*P* value
Block duration	28.52 ± 2.47	29.68 ± 2.44	0.11
Time to first request	3.9 ± (2.23)	8.6 ± (0.69)	< 0.001^a^
Intraoperative fentanyl consumption	62.5 ± (13.17)	60.18 ± (12.09)	0.5
Intraoperative vasodilator doses	1.05 ± (0.68)	1 ± (0.5)	0.78
postoperative vasodilator doses	0.75 ± (0.54)	0.72 ± (0.42)	0.84
Time to extubation	13 ± (4.23)	8.5 ± (3.34)	< 0.001^a^

^a^Statistical significance between two groups

**Fig. 1:**
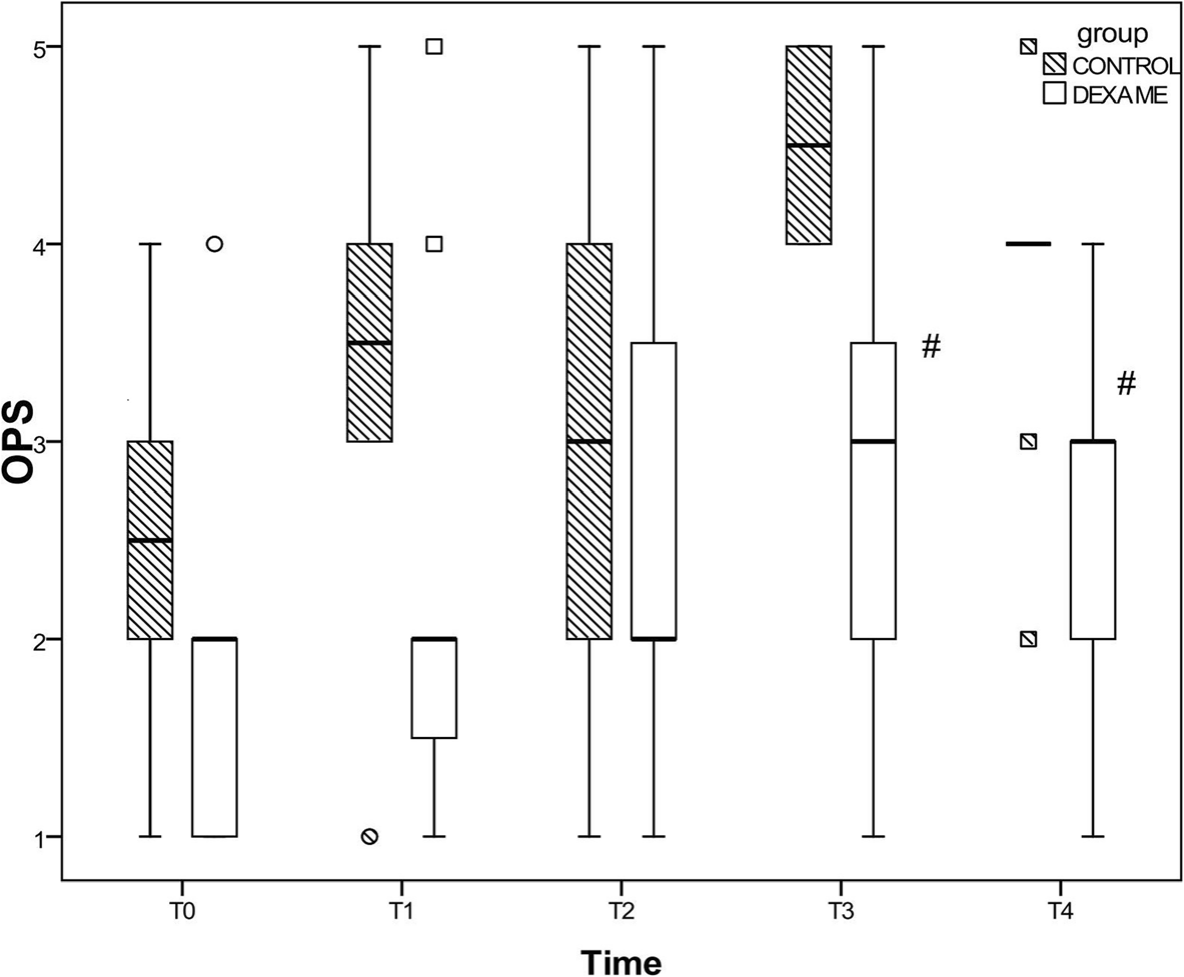
Median and IQR of OPS pain scores at different times. T0 = immediate postoperative, T1 = 4 h postoperative, T2 = 8 h postoperative, T3 = 12 h postoperative, T4 = 24 h postoperative. # *p* < 0.05

Regarding the OPS, the scores were lower in the dexamethasone group than in the control group, with a significant difference at T3 (12 h postoperatively) and T4 (24 h postoperatively) (Fig. 1**)**.

**Fig. 2:**
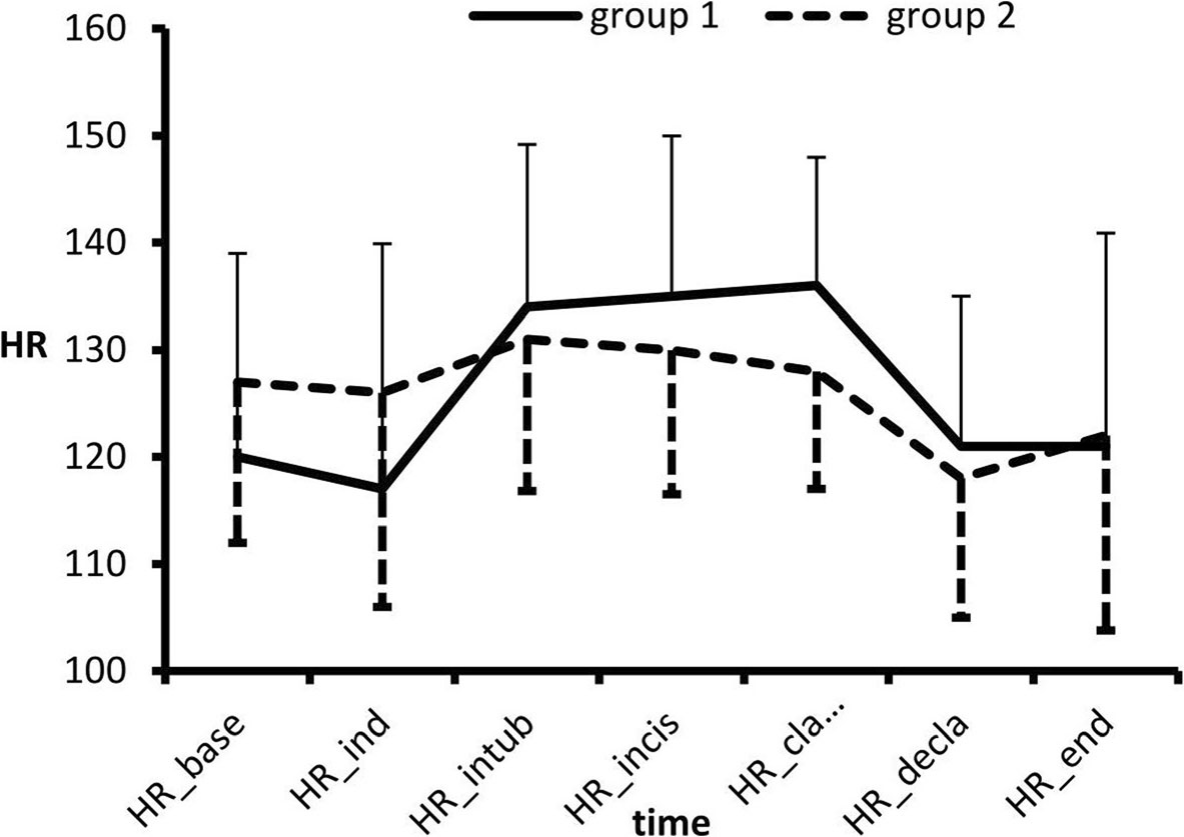
Mean ± SD of HR.

**Fig. 3:**
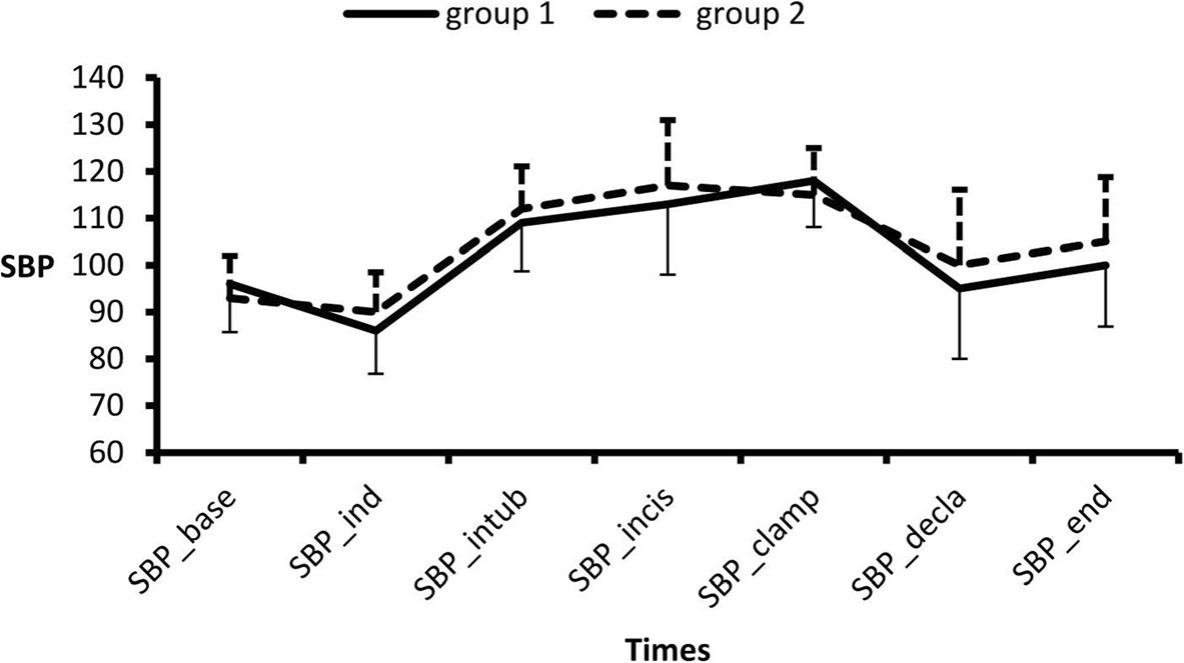
Means ± SD Systolic Blood Pressure at different study times. *P value< 0.05

There were no significant differences between the groups in the HR **(**Fig. 2**)** or systolic BP **(**Fig. 3**)** at any time.

Regarding complications, 5 patients in the control group experienced nausea and vomiting. None of our patients experienced pulmonary complications, respiratory depression, postoperative bradycardia or postoperative hypotension.

## Discussion

Our study demonstrated that the postoperative OPS was significantly lower at 12 and 24 h in the dexamethasone group than in the control group, demonstrating the prolonged analgesic effect of the combination of bupivacaine and dexamethasone in the paravertebral block. This finding is consistent with that of Sekhar et al. [12], who compared the VAS scores of patients in group C (bupivacaine + clonidine) and group D (bupivacaine + dexamethasone) at1 h (T 1),2 h (T2),3 h (T3),4 h (T4), 5 h (T5),6 h (T6), 12 h (T 12),24 h (T24) and 48 h (T48) postoperatively. The VAS scores were significantly lower in group D than in group C at all studied times.

Additionally, a study performed by ElFekyetal [13]. included 3 groups of paediatric patients undergoing infra-umbilical surgery with caudal block; fentanyl was used in the first group, dexamethasone was used in the second group, and dexmedetomidine was used in the third group. They found a significant decrease in the pain score after 3 h and in the number of patients who required analgesia during the first 6 h in both the dexmedetomidine and dexamethasone groups compared with the other group.

Our findings are in line with those of a study performed by Kim et al. [14], who investigated the effect of the addition of dexamethasone to ropivacaine in children who underwent orchiopexy and found significantly lower postoperative pain scores at 6 and 24 h post-surgery in the dexamethasone group.

ElKhafagy [15] evaluated pain intensity using the VAS score during the 1st 12 h postoperatively in 3 groups of adult patients undergoing lower abdominal surgery with epidural anaesthesia. The patients received bupivacaine (group B), bupivacaine +fentanyl (group BF) or bupivacaine + 4 mg dexamethasone (group BD). While the VAS scores of patients in groups BF and BD were comparable during the postoperative period, except at 12 h, their scores were significantly lower than those of patients in group B (*p* < 0.01).

Inadequate pain treatment after thoracotomy is associated with an increased incidence of postoperative complications in the form of retained lung secretions, atelectasis, and pneumonia. Additionally, thoracotomy pain can lead to increased thromboembolic complications and a prolonged hospital stay [16]. Paravertebral block has advantages that make it a reliable block for use in paediatric patients as it avoids autonomic dysfunction, which occurs in epidural blocks; additionally, in children, the paravertebral space is wider than the epidural space, which allows for easy identification of the space [17]. Thoracic paravertebral block is similar to thoracic epidural block regarding pain relief, hormonal responses and pulmonary function but has fewer side effects. There are some complications associated with paravertebral block, such as pleural puncture, vascular puncture and block failure, but the incidences of these complications are comparable to those of the complications of other regional techniques [18].

Contrary to the results of our study, Blanloeilet et al. [19] reported that epidural steroids (methylprednisolone) do not reduce postoperative pain after posterolateral thoracotomy. Possible explanations for this contradiction include different sampling sizes and the use of steroids with different potencies.

There was a significantly shorter time to the first analgesia request in the dexamethasone group than in the bupivacaine group. This finding is in line with those of Gautum et al. [20], who studied the effect of dexamethasone used with levobupivacaine in a paravertebral block on postoperative analgesia in patients undergoing thoracotomy. They found that the first dose of analgesia (sodium diclofenac) was needed 610 min postoperativelyin patients who received both dexamethasone and levobupivacaine versus 410 min postoperatively in patients who received levobupivacaine alone.

Our study showed no difference regarding intraoperative fentanyl consumption. Gautum et al. [20] compared intraoperative fentanyl consumption between dexamethasone with levobupivacaine versus levobupivacaine alone in a paravertebral block during thoracotomy. Fentanyl consumption was 112 μg in the dexamethasone plus levobupivacaine group and 115 μg in the levobupivacaine only group (*p* > 0.05).

Our study shows that the use of dexamethasone as an adjuvant facilitated early extubation; however, while the difference in extubation time was statistically significant at 4.5 h, this difference is not considered clinically significant in such patients. Moreover, while the intra- and postoperative vasodilator drug doses were lower in the dexamethasone group, there were no significant differences between the two groups.

AK Mohammed et al. [21] studied the possibility of early extubation after aortic coarctation repair in paediatric patients. They found that patients who underwent early extubation required a significantly higher nitroglycerine infusion dosage (4.267 ± 1.365 μcg/kg/min) to maintain their BP within the normal range for their age than patients who remained intubated at the end of the surgery (3.533 ± 1.147 μcg/kg/min). Jewell et al. [22] compared two groups of neonates undergoing aortic coarctation: patients in one group received general anaesthesia and ultrasound-guided paravertebral block, while those in the other group received general anaesthesia and opioid analgesia. They found significantly reduced postoperative analgesic consumption and earlier extubation times with the paravertebral block, but there were no significant differences in the intraoperative BP or the time of discharge.

There were no differences between the two groups regarding the HR or systolic BP. Sekhar et al. [12] showed no significant differences in the hemodynamic parameters between the dexamethasone and clonidine groups.

Two studies support our findings; both El Khafagy et al. [15] and El Feky [13] showed comparable hemodynamic measurements (mean arterial pressure (MAP), HR) in the different studied groups, with no significant differences throughout the intraoperative period.

In our study, five patients in the control group experienced postoperative nausea and vomiting versus no patients in the dexamethasone group. Our explanation is the use of dexamethasone reducing the incidence of nausea and vomiting and exerting an analgesic effect.

Ultrasound-guided techniques dramatically reduce the incidence of complications during block procedures. In addition, as an adjuvant, dexamethasone is safe and has many benefits without detected side effects.

A study by Fatma A A Zorob et al. [23] found that both the frequency and severity of complications were lower after paravertebral block than epidural block.

Pekka et al. [24] examined the effect of bupivacaine versus saline in a paravertebral block as a post-anaesthesia analgesic in mastectomy patients. They found that opioid consumption was reduced by 40% and that postoperative complications, especially nausea and vomiting, were reduced in the bupivacaine group.

Turkoz et al. [25] applied ultrasound-guided paravertebral block using bupivacaine in paediatric aortic coarctation repair after the induction of general anaesthesia. They found reduced hemodynamic parameters that exerted beneficial effects at site of the skin incision, reduced intra-and postoperative opioid consumption, and a greatly reduced incidence of postoperative nausea and vomiting, as well as a great improvement in respiratory function. The use of ultrasound increased the success of the procedure, and the analgesic effect was increased to 90–100%.

Our study was limited in that only paediatric patients undergoing one type of surgery were included. We recommend further investigation in patients of different ages undergoing various types of surgery, especially chest surgery. Also recommend catheterization for continuous anaesthesia and analgesia.

## Conclusion

The use of dexamethasone as an adjuvant in ultrasound-guided paravertebral block in paediatric patients undergoing aortic coarctation repair increased the duration of postoperative analgesia, with a prolonged time to the first request for analgesics, and allowed early extubation. Additionally, the use of dexamethasone was associated with a decreased incidence of postoperative complications. Adding dexamethasone in the paravertebral block did not affect the intraoperative hemodynamics or the vasodilator drug doses.

## Abbreviations

BP: Blood pressure
CBC: Complete blood count
HR: Heart rate
ICU: Intensive care unit
IM: Intramuscular
OPS: Objective pain score
OR: Operating room
VSD: Ventricular septal defect
